# Cosmetic penile enhancement surgery: a 3-year single-centre retrospective clinical evaluation of 355 cases

**DOI:** 10.1038/s41598-019-41652-w

**Published:** 2019-04-19

**Authors:** Alessandro Littara, Roberto Melone, Julio Cesar Morales-Medina, Tommaso Iannitti, Beniamino Palmieri

**Affiliations:** 1 Blumar Medica, Viale Vittorio Veneto 14, Milano, Italy; 2 Centro de Investigación en Reproducción Animal, CINVESTAV-Universidad Autónoma de CP 90000, AP 62, Tlaxcala, Mexico; 3 KWS BioTest, Marine View Office Park, Portishead, BS20 7AW United Kingdom; 4 Department of General Surgery and Surgical Specialties, University of Modena and Reggio Emilia Medical School, Surgical Clinic, Modena, Italy

**Keywords:** Anatomy, Testis

## Abstract

Men’s satisfaction and sexual function is influenced by discomfort over genital size which leads to seek surgical and non-surgical solutions for penis alteration. In this article we report the results of a retrospective study of 355 cases of cosmetic elongation, enlargement and combined elongation and enlargement phalloplasty. We found a significant improvement in length at rest, stretched length and circumference at rest at 2, 6 and 12 months post-surgical procedure (all p < 0.0001). 5-item International Index of Erectile Function (IIEF-5) was also increased at 12 months post-surgery compared to baseline (p < 0.0001). This was consistent with an IIEF-5 improvement of 6.74% compared to baseline. This study is clinically relevant due to the large cohort of patients included and because it is the first study to use an inverse periosteal-fascial suture not described previously as part of the surgical methodology.

## Introduction

Male genital image is correlated, albeit not in a necessarily linear manner^1^, to overall body image, psychosocial variables and sexual health^2^; in turn, sexual health is correlated to genital image^3^. Concern over genital endowment has archaic roots^4,5^. It typically emerges during adolescence^6,7^ and is triggered more by comparison among men than by the fear of not satisfying the partner^8^. Discomfort over genital size can influence satisfaction and man’s sexual function and push him to look for surgical and non-surgical solutions for penis alteration. We present a retrospective study of 355 cases of phalloplasty performed between 2012 and 2014.

### Penile size

The remarkable differences in the penile measurements reported by various authors can be explained by the methodological differences and the variety of the characteristics, even ethnic, of the populations studied (Tables 1 and 2). Furthermore, these measurements were rarely conducted on statistically adequate samples. The availability of regulatory data per defined population would be essential not only for diagnostic and therapeutic purposes, but also to reassure patients who display feelings of inadequacy^1,7,9,10^ and to manufacture correctly sized prophylactics^11^. Penis size is an anthropometric measurement^12^ and is correlated to anthropometric measurements such as height, weight and body mass index (BMI)^12,13^. These measurements are intercorrelated^13^ and they are polygenic traits subject to multifactorial influences^14^.

**Table 1:** Global published data of mean penile size (excluding self-reported measurements).

First author, year	Country	*N*	Age (years; range)	FPL (cm)	SPL (cm)	EPL (cm)	FPG (cm)	EPG (cm)
Loeb, 1899^50^	Germany	50	17–35	9.41				
Schonfeld, 1942^51^	USA	71	18–19		13.11		8.50	
		54	20–25		13.02			
Kinsey, 1948^52^	USA	2,770	20–59	9.07	15.05			
Aimani, 1985^53^	Nigeria	320	17–23	8.16			8.83	
Bondil, 1992^54^	France	905	17–91	10.07	16.74			
Da Ros, 1994^55^	Brasil	150				14.05		11.92 proximal 11.05 distal
Wessells, 1996^9^	USA	80	21–82	8.85	12.45	12.89	9.71	12.3
Smith, 1998^56^	Australia	184				15.71		
Chen, 2000^57^	Israel	55	21–78	8.03	12.05	13.06		
Ponchietti, 2001^12^	Italy	3,300	17–19	9.0	12.5		10.0	
Schneider, 2001^11^	Germany	111	18–19	8.60		14.48	9.68	
		32	40–68	9.22		14.48	9.02	
Sengezer, 2002^58^	Turkey	200	20–22		8.98			
Shah, 2002^59^	UK	104	17–84		13.0			
Spyropoulos, 2002^60^	Greece	52	19–38		12.18			
Son, 2003^61^	Korea	123	19–27	6.9	9.6		8.5	
Savoie, 2003^62^	USA	124	59.1 (avg.)	9.0	13.0			
Pereira, 2004^63^	Portugal	498	20:26	9.85	15.14		9.39	
Awwad, 2005^64^	Jordan	271	17–83	9.3	13.5		8.98	
		109	22–68	7.7	11.6	11.8		
Mehraban, 2007^26^	Iran	92	20–40		11.58		8.66	
Promodu, 2007^13^	India	500	18–60	8.21	10.88	13.01	9.14	
Kamel, 2009^65^	Egypt	949			12.9		8.9	
		78		11.2			8.8	
Nasar, 2011^14^	Egypt	1,000		8.37	13.77		10.48	
Khan, 2012^23^	Scotland	609	16–90	10.2	14.3			
Söylemez, 2012^66^	Turkey	2,276	18–39	8.95	13.98		8.89	
Chen, 2014^67^	China	5,196		6.5	12.9		8.0	
		311≈				12.9		10.5
Shalabi, 2015^68^	Egypt	2,000	22–40		13.84			
Veale, 2015^69^	UK	15,521	17–91	9.16		13.12	9.31	11.66
Habous, 2015^70^	Saudi Arabia	778	20–82			12.53/14.34	11.50	
Salama, 2016^71^	Egypt	239		7.4		11.8	8.7	11.3
Hussein, 2017^72^	Afghanistan	223		9.8	12.6			

FPL = Flaccid Penile Length; SPL = Stretched Penile Length; EPL = Erect Penile Length; FPG = Flaccid Penile Girth; EPG = Erect Penile Girth; (avg.) = average.

**Table 2:** Global published data of mean penile size (self-reported measurements only).

First author, year	Country	*N*	Age (range)	FPL (cm)	SPL (cm)	EPL (cm)	FPG (cm)	EPG (cm)
Richters, 1995^73^	Australia	156				15.99		
Bogaert, 1999^74^	USA	935	30 (avg.)	10.41	16.4	16.4	9.65	12.57
		4,187	30 (avg.)	9.83		15.6	9.40	12.19
Harding, 2002^75^	UK	312				15.25	12.55	
Schaeer, 2012^76^	Middle East	804				15.6		
Herbenick, 2013^77^	USA	1,661	17–91			14.15		12.23
Shaeer, 2013^78^	USA	1,133	52.38 (avg.)	13.1	15.6	16.3	10.6	

FPL = Flaccid Penile Length; SPL = Stretched Penile Length; EPL = Erect Penile Length; FPG = Flaccid Penile Girth; EPG = Erect Penile Girth; (avg.) = average.

## Materials and Methods

All methods and procedures were carried out in accordance with the principles contained in the Declaration of Helsinki.

### Patients

This study was registered on 04/04/2017 (ISRCTN number: ISRCTN60774878). 355 men participated in this retrospective clinical study. They came to our centre in Milan (Italy) for a cosmetic phalloplasty between 2012 and 2014 [cosmetic elongation (21), enlargement (33) and combined elongation and enlargement (301)]. The patients’ medical history was gathered and they underwent a medical examination that included an objective examination of the external genitals and the prostate, routine blood tests, basal penile ultrasound scan to verify the presence of nodules, plaques or lesions in the internal tissues of the penis and measurement of the length and circumference of the penis at rest (flaccid) and stretched. The stretched penis length (SPL) is considered a trustworthy approximation of the penis length during erection^1^. The 5-item International Index of Erectile Function (IIEF-5) is a validated diagnostic test that we administered to all the patients included in this study. All patients signed the informed consent to undergo the procedure and for the video to be published.

Measurement was always performed in the same room, by the same operator and using the same flexible measure after a brief introductory interview, performed to put the patient at ease. The measurement was performed before the ultrasound scan to avoid variations caused by changes in temperature. The measurement of the length was performed according to Mondaini *et al*.^7^. The length of the penis is defined as the linear distance along the dorsal side of the penis between the pubo-penile junction and the tip of the glans, either in the flaccid or stretched states. The circumference of the penis was measured at rest at mid-shaft. In all cases we found that the measurements were coherent with the morphometric values of reference of adult men according to Wessels and Ponchietti^9,12^ and this information was shared with the patients. After measuring height and weight using methods routinely employed in the clinical setting, the general medical examination continued with an in-depth interview conducted in order to investigate the patients’ motivations and expectations, discuss the foreseen method and the results and provide in-depth answers to the patients’ questions. A meeting between the patients and the anaesthetist occurred separately. At the end of the general examination, patients received instructions to be followed the night before and the morning prior to the surgical operation. In addition, we gave our availability to answer the patients’ questions at any time until the procedure took place. The information summarised in Table 3 was also discussed with all the patients during the general medical examination.

**Table 3:** Information regarding the phalloplasty discussed with the patients during their general examination.

Elements discussed by the physician with the patients during the general medical examination
(a) The estimated results given by our centre (+1.5–4.0 length, +20–35% circumference) refer to an increase between a minimum and maximum obtained from a historic average of all the patients operated both for elongation and enlargement. The availability of a vast collection of pre- and post-surgical photographs shown during the general examination confirmed such variability;
(b) it is possible that an increase cannot be achieved following the procedure and the achievable increase in each case can only be partially foreseen and depends on 1) the consistency and especially the depth of the suspensory ligament which can be overall evaluated sonographically (evaluation of the penopubic space superficially) concerning the elongation of the penis; 2) subjective variables such as metabolism and lifestyle which can increase or accelerate the reabsorption of the implanted fat concerning the enlargement of the penis;
(c) the increase acquired in terms of length is markedly more visible in conditions of flaccidity than in erection, with a ratio of about 3:1;
(d) occasionally, implanted fat can be subject to excessive reabsorption during the first three months after surgery and, in that case, if the patient wishes, a new definite transplant can be performed;
(e) in enlargement phalloplasty, the different consistency between the fat and the cavernous bodies causes a change in the tactile consistency of the penis; along the shaft, such change is progressive so that no “steps” are felt, and there is no variation in the quality of the erection or local sensitivity;
(f) after an elongation procedure, a slight change in the angle of erection can occur, more marked if the increase is significant (10–15 degrees);
(g) exceptionally, nodularity can occur in the implanted fat; such nodularity is, however, transitory and almost always resolves spontaneously.

The cosmetic phalloplasty candidate is a healthy and potent man with no congenital or acquired abnormalities or urogenital diseases. In this study, exclusion criteria were:

- coagulopathies, cardiopathies, neoplasies, chemo-radiotherapy, infections in progress, prior pelvic surgeries for urogenital conditions or trauma, severe systemic conditions and psychiatric conditions;
- unrealistic expectations; patients who requested results superior to those declared by the centre or who felt entitled to obtain the maximum penile increase within our historic series were excluded;
- revision surgery; patients requesting a re-operation because of the failure of a previous cosmetic phalloplasty were excluded;
- true hypoplasia (micropenis) defined as length <2.5 percentile points according to Mondaini^6^ (these patients were referred to an andrology centre);
- significant anxiety, distorted body image, a history of suicidal thoughts and/or attempted suicide linked to presumed genital inadequacy with psychogenic sexual dysfunction.

In line with data shown in the literature^2,5,7^, penis dimensions at rest were the most critical (78%) for patients but the circumference of the penis was more determinant than length (69%). This may depend, at least in part, on the concept that enlargement phalloplasty is less invasive than lengthening phalloplasty. The desire to increase both dimensions was the most frequent (82%); in many cases it was conditioned by the fear of losing the right penile proportions by intervening in only one aspect (66%) and it was probably facilitated by the advantages in terms of down-time connected with performing the two procedures simultaneously. The time that elapsed between the first examination and the surgical procedure was 2–6 months. Among the motivations for seeking this surgical procedure, the most frequently cited by patients were psychological discomfort in homosocial situations, discomfort towards women – almost always linked to one or more devaluing observations made during intimacy, the desire to “dazzle” women, the well-founded perception that genital size was incoherent with their body, the desire to improve an already generous natural endowment for narcissistic or professional reasons, the desire for better correlation or proportions between dimensions at rest and during erection and between length and girth and the desire to move from the lowest limits of the normal range towards the morphometric median. The most common concerns relative to the operation, which coincided with the patient’s expectations from the procedure, were: (a) the surgery being imperceptible (b) the preservation of the quality of erection and local sensitivity, (c) achievement of the mathematical average of the declared results, in terms of penis length and/or of the circumference and (d) the results being aesthetically impeccable.

### Anaesthesia

The choice of anaesthesia for cosmetic phalloplasty must be in line with the criteria of clinical adequacy, minimum invasiveness and rapid discharge. Among the different choices of anaesthesia, a vast array of scientific documentation^15^ exists to support the decided clinical advantages of sedation methods associated with local and loco-regional anaesthesia techniques. On the basis of such scientific support, we have opted for the following anaesthesia protocol:

#### Sedation

Premedication: Midazolam 0.04–0.05 mg/kg

Induction: Fentanyl 0.7–0.8 g/kg + Propofol 0.8–1.6 mg/kg

Maintenance: Propofol 0.3–0.5 mg/kg/hour

Only in rare cases (n = 6) it was necessary to use additional amounts of Propofol (0.5–0.8 mg/kg) and/or Fentanyl (0.4–0.8 g/kg) to guarantee adequate sedation.

#### Local anaesthesia

Anaesthesia in the pubic and penile region was executed by the surgeon using deep infiltration in the zone of the suspensory ligament of the penis and the cutaneous/sub-cutaneous zone affected by the surgical aggression:

Lidocaine 2%, 20 ml

Mepivacaine/carbocaine 2%, 10 ml (total solution 30 ml)

10 ml of the above mentioned solution was used in its pure form for cutaneous and deep peri-nervous infiltration, while the same was diluted in 230 ml of 0,9% sodium chloride with 1 mg epinephrine (1/250.000) for infiltration in the subcutaneous region where adipocytes will be harvested. In our experience, such procedure resulted to be fully ideal to allow surgical treatment, devoid of complications and major side effects, widely liked by patients and guaranteed brief protected discharge times (180 ± 30 minutes).

### Surgical procedure

#### Fat Harvesting and Purification

Prior to the operation, the patients were photographed while standing. The operation began after disinfection of the skin, with the harvesting of the adipose tissue. This was performed by explanting fat bilaterally from the thighs if the patient was tendentially thin and from the periumbilical region if the patient was normo-weight or overweight and from the suprapubic region if there was any localised adiposity. This latter area of harvesting permitted, in certain cases, the reduction of the suprapubic adipose panniculus (suprapubic lipectomy) rendering the point of insertion of the penis deeper and visually increasing the length of the external portion of the penis (see supplementary file).

Thereafter infiltration of the donor site was performed with a tumescent solution. After a few minutes of waiting, necessary to consolidate the vasoconstrictor effect of the epinephrine, adipose explant was performed using a thin cannula (2 mm) and a 10 cc Luer-lock syringe. The quantity of fat explanted varied from subject to subject on the basis of the volume to be filled, but it was never less than 80 ml. That volume was comprised of infiltration material which was then removed by decantation first and centrifugation later. Such a process of purification is of primary importance since it determines the percentage integration of fat in the penis. In our surgical centre we first performed the decantation through sedimentation of each 10 cc syringe in such a way as to put the harvested material through an initial process of purification. Each syringe was filled with fat again and each time the infiltration material was removed, repeating the decantation by sedimentation process many times. Once a seemingly stable mixture was obtained, the syringes of crudely purified fat underwent centrifugation for two minutes at 1000 rpm. Reducing the time and the number of rpms, with respect to the original Coleman’s technique which involves centrifugation for 3 minutes at 3000 rpm, the integrity of the adipose globules, whose integrity is in turn responsible for the good integration of the fat, was safeguarded. In the meantime, for the patients who received elongation phalloplasty, a 980 nm diode laser was used.

#### V-Y Plasty and Dissection of the Suspensory Ligament

**Figure 1:**
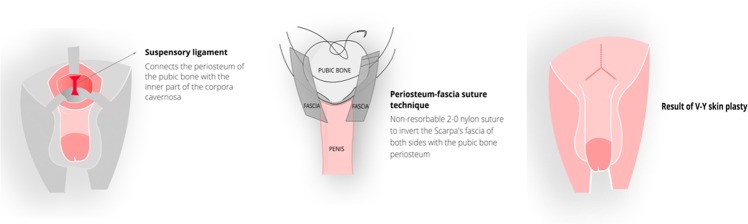
The inverse periosteal-fascial suture is intended to prevent the post-operative scar retraction of the dissected suspensory ligament. In order to reach and dissect the suspensory ligament, the Scarpa’s fascia (fundiform ligament) is first sectioned. Once the severing of the suspensory ligament is completed, a non-resorbable 2-0 suture is applied. It initially involves the left side Scarpa’s fascia, then the pubic bone periosteum in the deepest possible portion and then again the contralateral, right side Scarpa’s fascia. When tightening the suture knot, an introflection (inversion) of both bands towards the sloping point of the pubis is obtained. It thus fills the space formed by the section of the suspensory ligament and allows for the forward sliding of the penis. This technique prevents the post-operative retraction of the suspensory ligament, a frequent cause of surgical failure, and ensures a permanent and gratifying result.

The suprapubic area was incised using the inverted V technique (V-Y Plasty), which is more preferable than the Z technique or other techniques since it guarantees a better aesthetic result^16^ and is widely used in plastic surgery (Fig. 1). This was followed by a complete section of the suspensory ligament of the penis, taking care to adequately section the lateral ligaments as well. Only in this way it is possible to obtain the best achievable results. The suspensory ligament of the penis is a deep structure that joins the cavernous bodies of the penis to the pubic symphysis; its section entails the forward translation of the internal portion of the penis with the consequent increase in the length of the visible penile volume. In order to avoid post-surgical scar retraction of the ligament, inverse periosteal-fascial sutures were used. This technique ensured that the most superficial ligamentous tissues, which had been sectioned, were inverted into the newly formed cavity and then anchored with 2-0 nylon stitches in the deepest portion of the periosteum of the pubic symphysis. A first deep layer of suture was performed using a 3-0 slow resorption material suturing the ligament in a longitudinal direction. In effect, the ligament was initially sectioned horizontally and then sutured longitudinally thereby obtaining a postero-anterior increment in length that supported the increment obtained through the section of the deep ligaments. We used a technique similar to that employed by Brisson, 2001^17^. His technique allowed him to obtain a valid increase in the length of the external part of the penis and, at the same time, avoid scar-retraction phenomena that in the past nullified the increase obtained after a few weeks. Moreover, this quick and simple technique avoided the use of materials foreign to the organism, such as spacers of various kinds. A second layer of sutures was then performed always longitudinally using resorbable 3-0 sutures. Finally, the cosmetic closure of the cutaneous cut was performed using resorbable intradermal 4-0 sutures (V-Y plasty).

#### Fat Transfer

Once the penile elongation operation had been performed, the test-tubes containing the purified adipose material were extracted. They typically contained three layers: the most superficial was oily, the middle contained the purified fat and the lower was made up of blood and infiltration material^18^. The inferior and superior layers were eliminated and the purified material was implanted. Two mini-incisions of about 4 mm were made close to the pubo-penile junction at 10 o’clock and 2 o’clock respectively. The purified adipose material contained in the 10 cc syringes was decanted using a specific connector into 2.5 cc syringes better suited for the job. A blunt-tip cannula 2 mm in diameter was used for the implantation. The purified fat was then implanted into the subdartoic space taking care of the tunnel using the cannula and arranging the implant symmetrically. The space addressed was relatively avascular and, as a result, the formation of localized haematomas was rarely observed. In the few cases where hematomas were observed, bandaging was applied. Adverse events are summarised in Table 4. The quantity to be implanted varied considerably depending on the space to be filled, also considering that 30% of the implant would be resorbed within the first/second month. Therefore we decided to inject a modestly superior quantity to take into account its predicted partial resorption. At the end of the implantation, the surgical wound was closed and sutured using resorbable thread, a manoeuvre of manual “kneading” of the penis was then performed^19^ to aid in the uniform distribution of the implanted fat and finally a cohesive elastic bandage of adequate thickness was applied. The bandage has the important function of preventing the formation of crude asymmetries caused by posture and/or frequent erections during the first month after the operation. In fact, statistically at least 30 days are needed for the implant to be consolidated and the fat integrated and it is useful to limit the movement of the fat during this period using the elastic bandage. At the end of the operation a modestly compressive dressing was applied to the supra-pubic area and ice locally. The patient was discharged that evening with directions for medical therapy at home and adequately informed of the recovery period. In particular, the patient was urged to abstain from intense physical activity for 30 days and from sexual and masturbatory activity for 60 days. The duration of the operation was recorded from the moment of sedation until the final suture and it was about 80 minutes.

**Table 4:** Summary of adverse events.

Adverse events	PL + GE (N = 301) (n)	PL (N = 21) (n)	GE (N = 33) (n)	(N = 355) Total (%)
Loss of erectile function	0	0	0	
Decrease of erectile function (temporary)	2	1	0	0.008
Penile oedema	0	0	0	
Long-standing haematoma	2	1	1	0.011
Seroma	2	0	0	0.005
Dehiscence	0	0	N/A	
No increase in girth	0	N/A	0	
Fat loss (>30%)	15	N/A	6	0.059
Fat nodules, fat lumps	1	N/A	1	0.005
Fat migration	1	N/A	0	0.003
Sclerosing lipogranuloma	0	N/A	0	
Loss of sensation (mild)	3	0	2	0.001
Fibrosis	0	0	0	
Superficial infection	1	0	1	0.005
Deep infection	0	0	0	
Paradoxical penile shortening	0	0	N/A	
No increase in length	0	0	N/A	
Delayed wound healing	3	1	N/A	0.011
Penile deformity	0	0	0	
Penile asimmetry	1	N/A	1	0.005
Penile curvature	0	0	N/A	
Decreased erection angle (penile instability)	1	1	N/A	0.005
Hypertrophic wound scarring	2	1	N/A	0.008
Keloid	1	0	0	0.003
Scrotalization	0	0	0	
Disfiguring advancement of suprapubic hairy skin	2	0	N/A	0.005

PL = Penile Lengthening; GE = Girth Enhancement; N/A = not applicable.

#### Statistical analysis

Penis length at rest, stretched length and circumference data were analysed using a One-Way Analysis of Variance (ANOVA) with Dunnet’s *post-hoc* test for comparison of each time point with baseline. IIEF-5 data were analysed using an unpaired two-sample Student’s *t*-test. All the data are presented as mean ± standard error of the mean (SEM). A p < 0.05 was considered significant.

## Results

**Figure 2:**
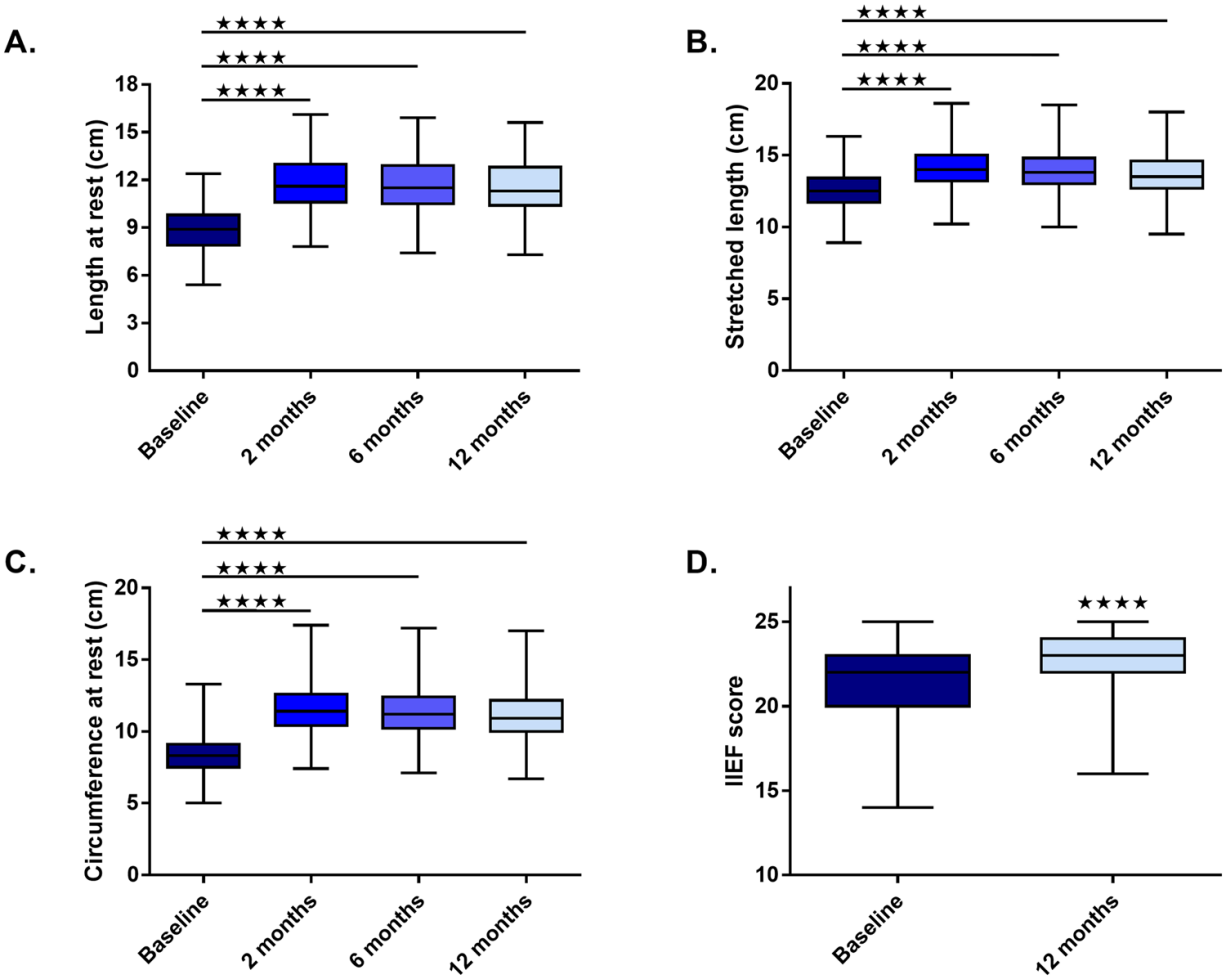
Cosmetic phalloplasty significantly improves penis length at rest (**A**), stretched length (**B**), circumference at rest (**C**) and 5-item International Index of Erectile Function (IIEF-5) score (**D**) at 2, 6 and 12 months post-surgical procedure. Line represents median. Four stars indicate statistical significance (p < 0.0001).

The baseline characteristics of the patients are summarized in Table 5. Following the surgical procedure, length at rest significantly increased at 2 (11.6 ± 0.08), 6 (11.5 ± 0.09) and 12 months (11.4 ± 0.1), compared to baseline (8.8 ± 0.07) (all p < 0.0001, respectively) (Fig. 2A). Stretched length significantly increased at 2 (14.02 ± 0.07), 6 (13.7 ± 0.08) and 12 (13.5 ± 0.09) months, compared to baseline (12.4 ± 0.06) (all p < 0.0001) (Fig. 2B). Circumference at rest significantly increased at 2 (11.5 ± 0.09), 6 (11.36 ± 0.09) and 12 (11.06 ± 0.1) months, compared to baseline (8.3 ± 0.06) (all p < 0.0001) (Fig. 2C). IIEF-5 increased at 12 months (23 ± 0.08) compared to baseline (21.5 ± 0.08) (p < 0.0001; 6.74% improvement) (Fig. 2D).

**Table 5:** Baseline descriptive statistics of patients’ demographics.

	Age (years)	Weight (kg)	Height (cm)	Baseline IIEF-5	Baseline length at rest (cm)	Baseline stretched length (cm)	Baseline circumference at rest (cm)
Number of values	355	355	355	327	354	355	354
Minimum	19	56	167	14	5.4	8.9	5
25% Percentile	29	68	173	20	7.9	11.7	7.5
Median	36	74	178	22	8.9	12.5	8.3
75% Percentile	46	81	181	23	9.8	13.4	9.1
Maximum	63	99	192	25	12.4	16.3	13.3
Mean	38.08	75.13	177.4	21.5	8.882	12.45	8.377
Std. Deviation	10.81	8.969	5.126	2.41	1.362	1.314	1.213
Std. Error of Mean	0.5737	0.476	0.2721	0.1333	0.07237	0.06974	0.06445
Lower 95% CI of mean	36.95	74.2	176.8	21.24	8.739	12.31	8.25
Upper 95% CI of mean	39.21	76.07	177.9	21.76	9.024	12.58	8.504
Sum	13519	26672	62962	7031	3144	4419	2965

IIEF-5 = 5-item International Index of Erectile Function.

## Discussion

We found that cosmetic phalloplasty significantly improves length at rest, stretched length, circumference at rest and IIEF-5 score at 2, 6 and 12 months post-surgery.

Hypoplasia of the penis is associated with medical conditions which include low flow priapism^20^, Peyronie’s disease^21^, congenital abnormalities^22^, erectile dysfunction^23^ and surgical conditions such as radical prostatectomy/radiotherapy for prostatic carcinoma^24–26^ and surgical correction of Peyronie’s disease^27^. Evidence shows cases of apparent hypoplasia distinguishing it from (a) “hidden” penis, secondary to the presence of abdominal fat or the cutaneous relaxation of the abdomen^28^ and (b) a “buried” penis where the penis shaft is beneath the suprapubic skin as a result of obesity and/or radical circumcision^29^.

Associated with a cutaneous V-Y plasty, ligamentolysis is the main and most common method of surgical elongation of the penis^19,29–34^. Omission of the cutaneous plasty contrasts the result achieved from the release of the ligament because it impedes the advancement of the shaft^35^. Detachment of the suspensory ligament and the pubic symphysis, which is obtained through ligamentolysis, causes a forward movement of the cavernous bodies and allows the penis to reach its maximum extracorporeal projection. The elongation is considered purely apparent (“apparent lengthening” vs “genuine lengthening”) since the length of the penis remains unvaried; such elongation is in fact significantly more visible at rest than during erection^36^. Nevertheless, the operation produces a visible and available increase in the length of the penis as expected by the patient.

Several techniques have been proposed in order to impede retraction of the sectioned ligament and therefore nullify the surgical result. They include positioning of the fat obtained from the spermatic funniculi between the suspensory ligament and the pubic symphysis^19,37^, use of silicone spacers^38^, the application of weights^38^ and postsurgical penile stretching^39^. In a previous study, the post-surgical use of extensors, for at least three consecutive months, resulted in an increase of length of no more than 1.3 cm^40^.

The growing demand for autologous fat transplant (AFT) beginning at the end of the 80 s is linked to the advent of liposuction. The current methods of fat transfer were popularised and extensively described by Sydney Coleman^18,41,42^ who in 1986 began to transplant fat in iatrogenic deformities from liposuction and subsequently in the face. AFT is today a widely tested procedure, appreciated by patients and very widespread among plastic surgeons even for reconstructive surgery^43–49^ despite no consensus has been reached regarding the best technique or its success rate.

The fat injection is the most common technique of penile girth enhancement. The fat harvested from the patient is implanted into the subdartoic space with the objective to symmetrically and uniformly increase the circumference of the penis^29^.

The inhomogeneities of the surgical techniques and the selection criteria of the patients render it difficult to compare the results obtained by our centre with those found in the literature and reported from other clinics (an overview of surgical techniques employed for phalloplasty and results obtained is summarised in Table 6).

**Table 6:** Overview of surgical techniques employed for phalloplasty and results obtained.

First author, year	N	Method	Follow-up (months)	Gain in Length (cm)		Gain in Girth (cm)	
				***flaccid***	**erect**	***flaccid***	**erect**
Austoni, 2002^79^	39	Autologous safenous graft	9				1.1–2.1 (∅)
Perovic, 2006^34^	204	Biodegr. scaffolds coated w/augologous fibroblasts	24 (N = 84)			*3*.*15*	
Shaeer, 2006^80^	1	Superficial circumflex iliac artery island flap	6			*9*.*5*	8.5
Bin, 2009^81^	20	Saphenous grafts, PTFE artificial vessel patches	1–5 years			*1*.*0–2*.*3*	1.5–3.0
Jin, 2011^82^	69	Biodegr. scaffolds coated w/autologous fibroblasts	1,3,6			*4*.*01*	2.92
Alei, 2012^83^	69	Porcine dermis graft	6.12			*3*.*1*	2.4
*Wessels, 1996* ^84^	12	*SLD (N* = *12)* + *VYP*, *FI (N* = *10)*					
*Alter, 1997* ^85^	30	*SLD* + *VYP*, *FI*					
*Klein, 1999* ^6^	*58¹*	*SLD (N* = *10)*, *FI (N* = *1)*, *SLD* + *DFG (N* = *6)*, *SDL*, *FI (N* = *41)*	*12*.*2*	*3*.*0*	*0*.*76*	*2*.*52*	*2*.*03*
Roos, 1994^8^	260	SLD, Y(Z/M flap)	4 (N = 100)	*4*.*0*			
Shirong, 2000^86^	52	SLD + scrotal flap/skin graft, VYP	6 (N = 20)	*3*.*5–6*.*5*			
Spyropoulos, 2005^31^	11	SLD, VYP (N = 5) SLD, DFG (N = 3) SBL, SLD (N = 2)		*1*.*6*		*2*.*3/2*.*6*	
Li, 2006^38^	42	SLD (N = 42) + silicone spacer (N = 27), VYP (N = 17)	16	*1*.*3 (stretched)*			
Panfilov, 2006^19^	88	SDL, FI (N = 31) FI (N = 57)	12	*2*.*42*		*1*.*0–4*.*0*	
Mertziotis, 2013^87^	82	SLD + VYP, DFF (N = 35)	12	*1*.*92*		*2*.*21*	
		circumcision ligamentolisys, DFF (N = 47)		*2*.*11*		*2*.*0*	
Monreal, 2015^40^	259°	SLD, FI (N = 148) FI (N = 127)	6 (N = 160) 12 (N = 87)	*3*.*1* *3*.*2*		*1*.*7* *1*.*6*	
Xu, 2016^88^	23	SLD + DFT		*2*.*27*		1.67	

DFF = dermal fat flap; DFT = dermal fat transfer; FI = fat injection; SBL = suprapubic lipectomy; SLD = suspensory ligament dissection; VYP = V-Y plasty.

¹Data obtained from questionnaires administered in several surgical centers by at least 10 different surgeons.

°Total number of procedures: 275.

In our experience, cosmetic phalloplasty has evolved in time moving in a direction of increased safety. The substitution of silicone spacers with inverse periosteal fascial sutures, which we have already described, and the use of autologous fat have marked the end of rare but significant complications that in the past led to reoperation. At the moment, we employ a surgical technique that keeps complications to a minimum and and results in great patients’ satisfaction. Patients who undergo combined elongation and girth enhancement phalloplasty are particularly satisfied compared to those who undergo a single operation which is probably linked to the availability of an overall greater penile volume^40^. In Italy, there is no validated test for the measurement of patients’ satisfaction in cosmetic penoplasty and the absence of a measurement of patients’ satisfaction is also a limitation of our study.

In line with other authors, we believe that, even in its relative simplicity, cosmetic phalloplasty requires a profound knowledge of anatomy and surgical technique and that the selection of candidates is a fundamental and essential element together with scrupulous gathering of information regarding not only the operation and the obtainable results, but also post-surgical conduct since resuming of sexual activity prior to 60 days after the operation can compromise the results.

While confirming that cosmetic phalloplasty very rarely produces spectacular results and that there is an objective necessity to improve the stability of the fat in time, we retain that the data from our centre show that the surgical technique we utilise is safe, repeatable and produces concrete and measurable results. Finally the operation, last resort to improve the patient’s discomfort, can considerably improve the patient’s self-esteem and improve the quality of his sex life and, in turn, his relationships.

## Conclusions

The limited literature regarding cosmetic phalloplasty consists of studies performed using diverse surgical techniques and candidate selection criteria which include patients who should in fact be excluded (e.g. men with psychiatric conditions, namely body dysmorphic disorder) or whose existing conditions (e.g. failure of previous phalloplasty and trauma) make it impossible to compare results. If we consider the lack of universally shared morphometric values, we see how this niche of cosmetic surgery suffers from an inevitable lack of methodological rigour. In the present study we show the efficacy of cosmetic phalloplasty in a large cohort of patients up to 1-year follow-up. In addition, we describe in detail inclusion and exclusion criteria for patient selection and technical aspects of our surgical procedure which ensure reproducibility of our findings and should be adopted in future clinical studies of cosmetic phalloplasty. We are confident that this study will encourage other authors to publish their experiences with cosmetic phalloplasty and that the method we have described in this article will contribute to the consolidation of a standard for this type of surgery.

## Supplementary information

Phalloplasty video
